# Effects of temperature and shear on the structural, thermal and pasting properties of different potato flour

**DOI:** 10.1186/s13065-020-00670-w

**Published:** 2020-03-23

**Authors:** Ke Zhang, Yang Tian, Chenglong Liu, Wentong Xue

**Affiliations:** College of Food Science & Nutritional Engineering, China Agricultural University, Beijing, China

**Keywords:** Potato flour, Thermodynamics, Pasting properties, Structure, Texture

## Abstract

**Background:**

The properties of potato flour will be different due to different processing parameters, which will affect their processing adaptability. In this paper, different potato flour were investigated to determine viscoelastic properties and structural transformation using thermodynamics, rheological and spectrum methods. Potato flour was prepared by drying at different temperature after soaking in citric acid, microwave and steamed respectively. The treated samples were dried by hot air and then compared with the freeze-dried potato flour. Four kinds of potato flour showed different properties after shearing at high temperature.

**Results:**

Differential scanning calorimetry (DSC) results revealed that potato flour with low gelatinization had lower enthalpy and faster melting process than freeze-dried potato powder. RVA and texture results showed that potato flour with low gelatinization had the best retrogradation property and the stable gel. X-ray diffraction (XRD) patterns revealed that the crystalline properties of different potato flour after shearing at high temperature were the same. In addition, low gelatinization potato flour presented a crystalline structure or strong internal order. Fourier-transform infrared spectroscopy (FTIR) spectra showed that high temperature and shearing mainly caused δ-deformation of O–H in intact potato granules.

**Conclusion:**

Freeze drying and hot air drying at low temperature made potato flour had better gel stability than microwave and steamed treatment. Hot air drying at low temperature made potato flour had good retrogradation after hot shearing, which was more conducive to the formation of hot-processed products.

## Introduction

Potato (*Solanum tuberosum* L.) is an annual herb of Solanaceae, its fresh tubers can be eaten after simple processing. As one of the world’s major food crops, potato contains ascorbic acid, phenolic substances and other important active ingredients [1, 2]. However, high water content and metabolic activity lead to short shelf life and high storage cost, and limit the promotion of potatoes. Processing fresh potatoes into dried whole flour or starch can effectively solve this problem. Potato flour is one of dehydrated potato products. Granular, chip or powder products are prepared with fresh potatoes. The traditional processing technology includes cleaning, peeling, selection, slicing, dehydration and drying [3, 4]. The quality of potato flour is affected by processing method and parameters. Several methods have been used in potato flour by various authors to characterize the relationship between processing technology and product properties.

Browning of fresh potato during processing is an important factor affecting the quality of dry potato flour, which can be divided into enzymatic browning and non-enzymatic browning. Enzymatic browning makes potatoes murky grey, and non-enzymatic browning makes them yellowish-brown. In production, browning can be prevented by heat-treated or adding color fixative at room temperature. Vitamin C, citric acid and phytic acid are in common use in color protection operation. Wang et al. [5] found that enzyme inhibitor (vitamin C, citric acid and Na_2_SO_3_) could significantly inhibit the activity of polyphenol oxidase (PPO). When vitamin C was 0.35%, citric acid was 1.2% and Na_2_SO_3_ was 0.25%, the inhibitory effect on PPO was the best. Zhao et al. [6] used 0.03% vitamin C, 0.35% phytic acid, 0.25% citric acid and 0.03% l-cysteine to remarkably inhibit the browning of potato powder.

High temperature can inactivate polyphenol oxidase, and thus inhibit browning. However, excessive heat treatment or mechanical shear stress can lead to cell wall degradation and free state of starch and other nutrients in potato tubers. The physical properties of the product are difficult to preserve, its dispersibility and rehydration are not good, and it does not have the characteristics of fresh potatoes. Li et al. [7] obtained low starch gelatinization degree (14.52%) by flash drying technique. Binner et al. [8] studied the effect of cooking at 70 °C and 100 °C on the cell structure of potato and sweet potato. The results showed that the product had good powdery properties after cooking at 100 °C for 10 min. After cooking at 70 °C, β-amylase degraded starch into oligomers with small molecules and escaped from cells, which resulting in hard and fragile potato tubers. Kim et al. [9] used pectinase to treat snowflake pollen and found that the cell morphology changed. The retrograde starch granules are encapsulated by parenchyma cells, which restricts the outflow of starch molecules and reduces the outflow rate of starch. This method improves the ability of cell to resist damage by changing morphological structure.

Despite several studies on the physicochemical, rheological, and thermodynamics properties of potato flour have been reported earlier in several studies [3, 10–12]. The present work aims to contribute to knowledge about the effects that high temperature shear on four kinds of potato powder and their viscoelastic properties and structural transformation. This aim was achieved by collecting and analyzing DSC and RVA results, FTIR spectra and XRD patterns. Combining with the results of this study, suitable potato flour can be screened out for food with special quality requirements.

## Results and discussion

### Potato flour characterization

#### Degree of gelatinization

Hot air, microwave and steam change the temperature, moisture and particle structure of potato flour, which resulted in different degree of starch gelatinization (DG) of the product [13]. The DG (%) of potato flours were as follows: low gelatinization potato flour (LGPF): 27.73 ± 0.86; microwave potato flour (MPF): 94.24 ± 2.85; steamed potato flour (SPF): 95.63 ± 1.98.

#### Amylose content, swelling power and light transmittance

**Fig. 1:**
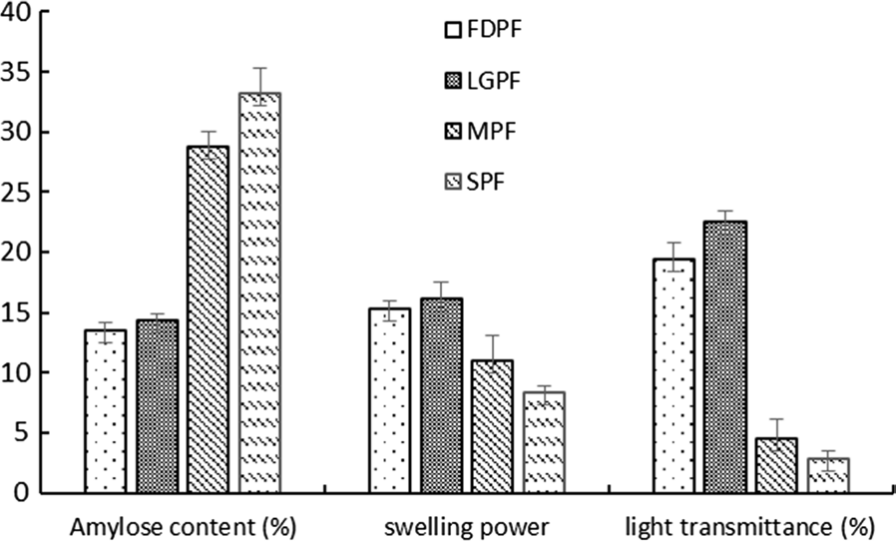
Amylose content, swelling power and light transmittance of potato flour. *FDPF* freeze-dried potato flour, *LGPF* low gelatinization potato flour, *MPF* microwave potato flour, *SPF* steamed potato flour

As shown in Fig. 1, the amylose content, swing power and light transmittance of FDPF and LGPF were close and lower than that of MPF and SPF, indicating that the starch particles in LGPF were less damaged, and the damage of microwave to potato starch was slightly less than that of steaming.

#### Particle size and distribution analysis

The potato flour microparticles were prepared by suspension sampling technique. The results obtained for particle size and size distribution were given in Table 1. For freeze-dried potato flour (FDPF) sample, about 90% of the microparticles were lower than 41.83 μm and 10% of the particles were lower than 6.34 μm. Particle size distribution of FDPF concentrated at 21.43 μm. The geometric mean of the particle size was found to be 22.76 μm with a median size of 17.93 μm. Freeze-drying can weaken the binding of molecules, resulting in small particle size in suspension.

**Table 1:** Microparticles size distribution

Potato flour	d_10_ (μm)	d_50_ (μm)	d_90_ (μm)	Mean (μm)	Mode (μm)	C.V. (%)
FDPF	6.34 ± 0.08a	17.93 ± 0.32a	41.83 ± 1.51a	22.76 ± 1.04a	21.43 ± 0.23a	81.83 ± 2.07c
LGPF	29.54 ± 0.09b	213.7 ± 4.3c	514.4 ± 6.42c	240.73 ± 3.34c	391.38 ± 0.34c	79.4 ± 0.36b
MPF	33.61 ± 0.14c	162.03 ± 1.17b	471.23 ± 2.21b	210.27 ± 1.17b	185.49 ± 0.08b	83.63 ± 0.45c
SPF	48.37 ± 0.41d	252.03 ± 0.95d	526.13 ± 3.69d	273.8 ± 1.44d	356.48 ± 0.45d	65.97 ± 0.42a

*FDPF* freeze-dried potato flour, *LGPF* low gelatinization potato flour, *MPF* microwave potato flour, *SPF* steamed potato flour

Microparticles prepared from LGPF showed a mean size of 240.73 μm with a median size of 213.7 μm. About 90% of the microparticles were lower than 514.4 μm and 10% of the particles were lower than 29.54 μm. Particle size distribution of FDPF concentrated at 391.38 μm. A large amount of water in the sample before hot air drying was beneficial to enhance the binding force between molecules, leading to the formation of large masses.

Microparticles prepared from MPF showed a mean size of 210.27 μm with a median size of 162.03 μm. About 90% of the microparticles were lower than 471.23 μm and 10% of the particles were lower than 33.61 μm. Particle size distribution of MPF concentrated at 185.49 μm. After microwave irradiation, the water content in potato chips was decreased, and the binding and aggregation of molecules was weakened.

Microparticles prepared from SPF showed a mean size of 273.8 μm with a median size of 252.03 μm. About 90% of the microparticles were lower than 526.13 μm and 10% of the particles were lower than 48.37 μm. Particle size distribution of SPF concentrated at 356.48 μm. Similar to LGPF, a large amount of moisture during drying led to an increase in the particle size of the powder.

According to the value of C.V., the particle size distribution of SPF was the narrowest, followed by LGPF, and FDPF and MPF were the widest.

#### SEM and optical microscope analysis

**Fig. 2:**
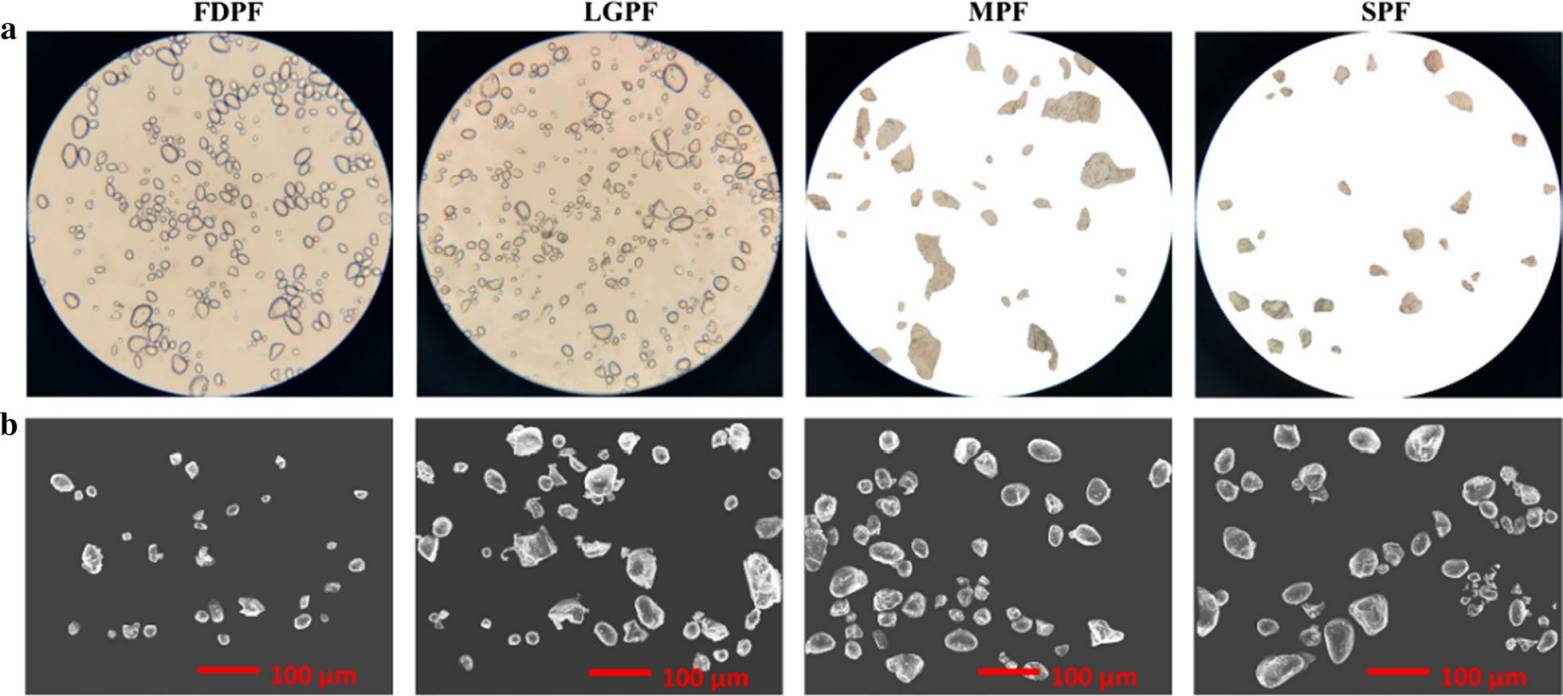
Scanning electron microscopy and optical microscopic images. **a** Optical microscopic photos of different potato flour; **b** scanning electronic microscopic photos of different potato flour. *FDPF* freeze-dried potato flour, *LGPF* low gelatinization potato flour, *MPF* microwave potato flour, *SPF* steamed potato flour

Figure 2 shows the optical microscope and scanning electron images of four different potato flour. Granule shape was retained for the FDPF and LGPF. The central point of FDPF can be clearly observed (Fig. 2a), and the surface of MPF and SPF was eroded, due to the gelatinization of potato starch. However, aggregation phenomenon, highly ordered fragments with compact structures and irregularly-shaped surfaces were observed in images of MPF and SPF (Fig. 2a, b).

#### Texture analyses

The gel forming ability of MPF and SPF was weak and not suitable for the detection of texture analyzer. Table 2 presents the texture of FDPF and LGPF. Hardness, adhesiveness, springiness and chewiness of LGPF were significantly higher than FDPF. The result was attributed to its stronger regeneration ability.

**Table 2:** Texture of potato flour paste

Paste	Hardness 1 (N)	Adhesive force (N)	Adhesiveness (N mm)	Hardness 2 (N)	Cohesiveness	Springiness (mm)	Chewiness (mJ)
FDPF	1.69 ± 0.06	0.23 ± 0.01	4.6 ± 0.06	1.58 ± 0.06	0.77 ± 0.01	1.92 ± 0.01	2.5 ± 0.13
LGPF	2.07 ± 0.06	0.15 ± 0.01	6.67 ± 0.27	1.96 ± 0.05	0.77 ± 0.01	2.54 ± 0.05	4.01 ± 0.2

### DSC analysis

No peak of MPF and SPF image was found in the DSC results. The gelatinization properties of FDPF and LGPF were observed by DSC, and the results are shown in Table 3. The gelatinization temperature parameters were represented by To (onset temperature), Tes (endset temperature), Tp (peak temperature), Ts (start gelatinization temperature), Te (end gelatinization temperature), and ΔH_G_. As can be seen from Table 3, Ts, Te, Tp, and To of LGPF were greater than that of FDPF. The result implied that LGPF need more energy than FDPF to start gelatinization and onset. According to ΔH_G_, the gelatinization process of LGPF spend less thermal energy than that of FDPF.

**Table 3:** Thermal characteristics analysis of FDPF and LGPF

Potato flour	Ts (°C)	Te (°C)	Tp (°C)	To (°C)	Tes (°C)	ΔH_G_ (mJ)
FDPF	60.3 ± 0.35	75.6 ± 1.02	67.5 ± 0.23	62.17 ± 0.18	73.74 ± 0.45	-25.16 ± 0.54
LGPF	61.98 ± 0.2	76.9 ± 0.35	69.35 ± 0.57	65.35 ± 0.33	73.85 ± 0.74	-15.94 ± 1.6

Values are the mean ± standard deviation

*Ts* start gelatinization temperature, *Te* end gelatinization temperature, *Tp* peak temperature, *To* onset temperature, *Tes* endset temperature, *ΔH*_*G*_ gelatinization enthalpy change

### RVA analysis

The pasting profiles of potato flour are shown in Table 4. The peak viscosity and breakdown value of FDPF were the largest. This observation could suggested that the binding among molecules is strong but easy to be destroyed during pasting for FDPF. The trough viscosity, final viscosity, setback value, peak time and pasting temperature of LGPF were the highest. These results implied that LGPF need the most energy during pasting, and have strong shear resistance and molecular recombination ability. The peak time and gelatinization temperature of MPF were the smallest, which indicated that MPF was most prone to pasting. The viscosity value of SPF was the lowest, likely due to that the shear resistance of SPF was the weakest.

**Table 4:** Pasting properties of different potato flour

Potato flour	Peak viscosity (cP)	Trough viscosity (cP)	Breakdown (cP)	Final viscosity (cP)	Setback (cP)	Peak time (min)	Pasting temperature (°C)
FDPF	5653 ± 72.13^d^	3078 ± 43.84^c^	2575 ± 28.28^d^	3948.5 ± 40.31^c^	870.5 ± 3.54^b^	3.87 ± 0^b^	66.78 ± 0.04^b^
LGPF	4886 ± 117.38^c^	4193.5 ± 103.95^d^	692.5 ± 13.44^b^	5566.5 ± 139.3^d^	1373 ± 35.36^c^	5.53 ± 0^d^	70.08 ± 0.11^c^
MPF	2497 ± 45.26^b^	1292.5 ± 17.68^b^	1204.5 ± 27.58^c^	2160.5 ± 36.06^b^	868 ± 18.39^b^	3.6 ± 0.1^a^	50.1 ± 0^a^
SPF	1189.5 ± 14.85^a^	983 ± 16.97^a^	206.5 ± 2.12^a^	1382 ± 25.46^a^	399 ± 8.49^a^	4.94 ± 0.09^c^	66.83 ± 0.11^b^

Breakdown (BD, the difference between the peak viscosity and the trough viscosity). Setback (SB, the difference between the trough viscosity and the final viscosity). Values are the mean ± standard deviation. Means in the same column having a different superscript are significantly different (*p < 0.05)

### XRD analysis

**Fig. 3:**
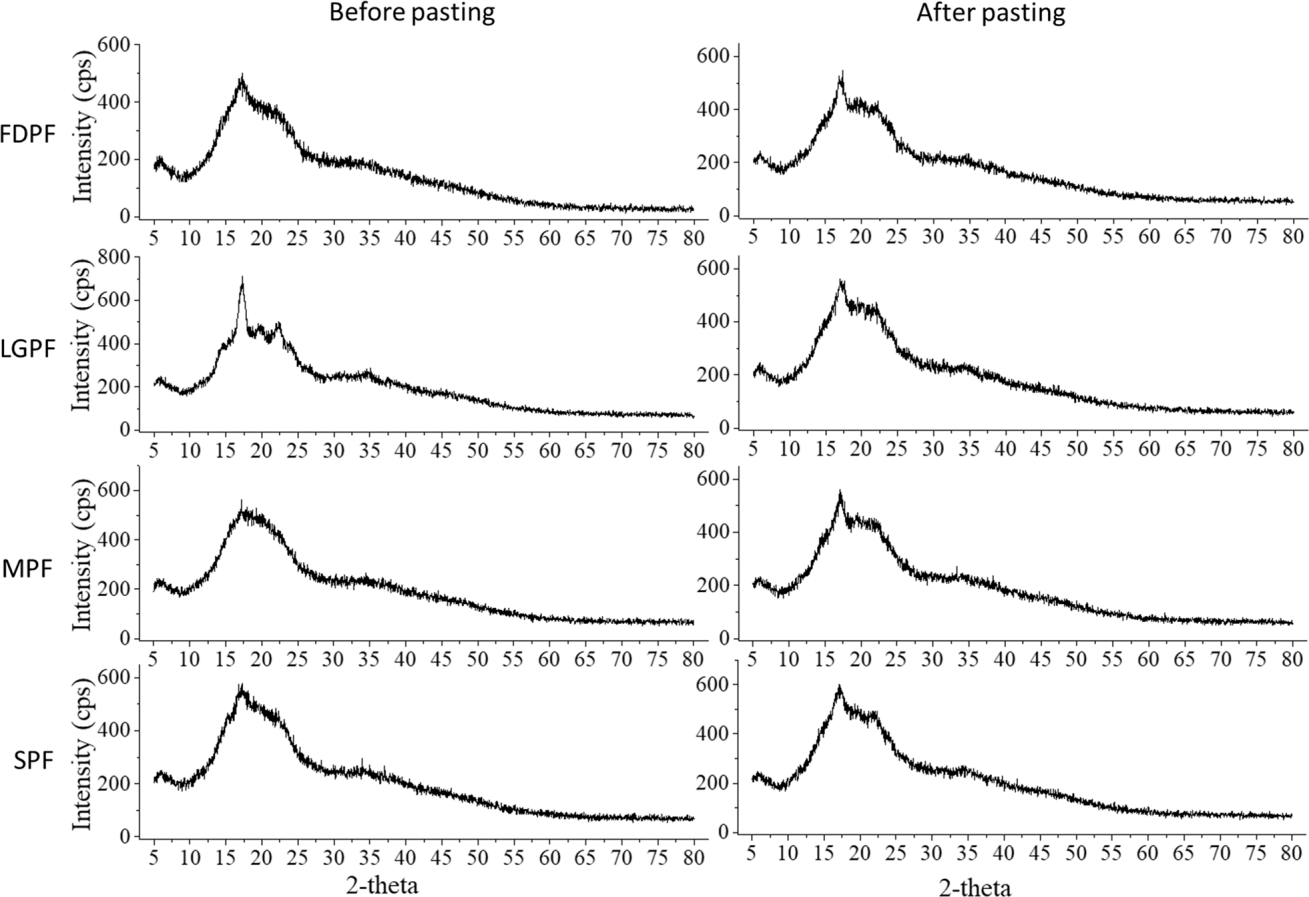
X-ray diffraction patterns of potato flour and their paste

Figure 3 shows XRD patterns for potato flour and their paste. There are similar characteristic diffraction peaks among potato flour paste. XRD of FDPF, LGPF and paste showed a sharp peak centered at about 16°, indicating their highly crystalline nature [14]. The X-ray powder diffraction pattern of MPF showed broad amorphous features. Microwave and steam decreased the amount of crystalline, increased the amount of the amorphous lamellar region, and therefore resulted in broad peak.

### FTIR analysis

**Fig. 4:**
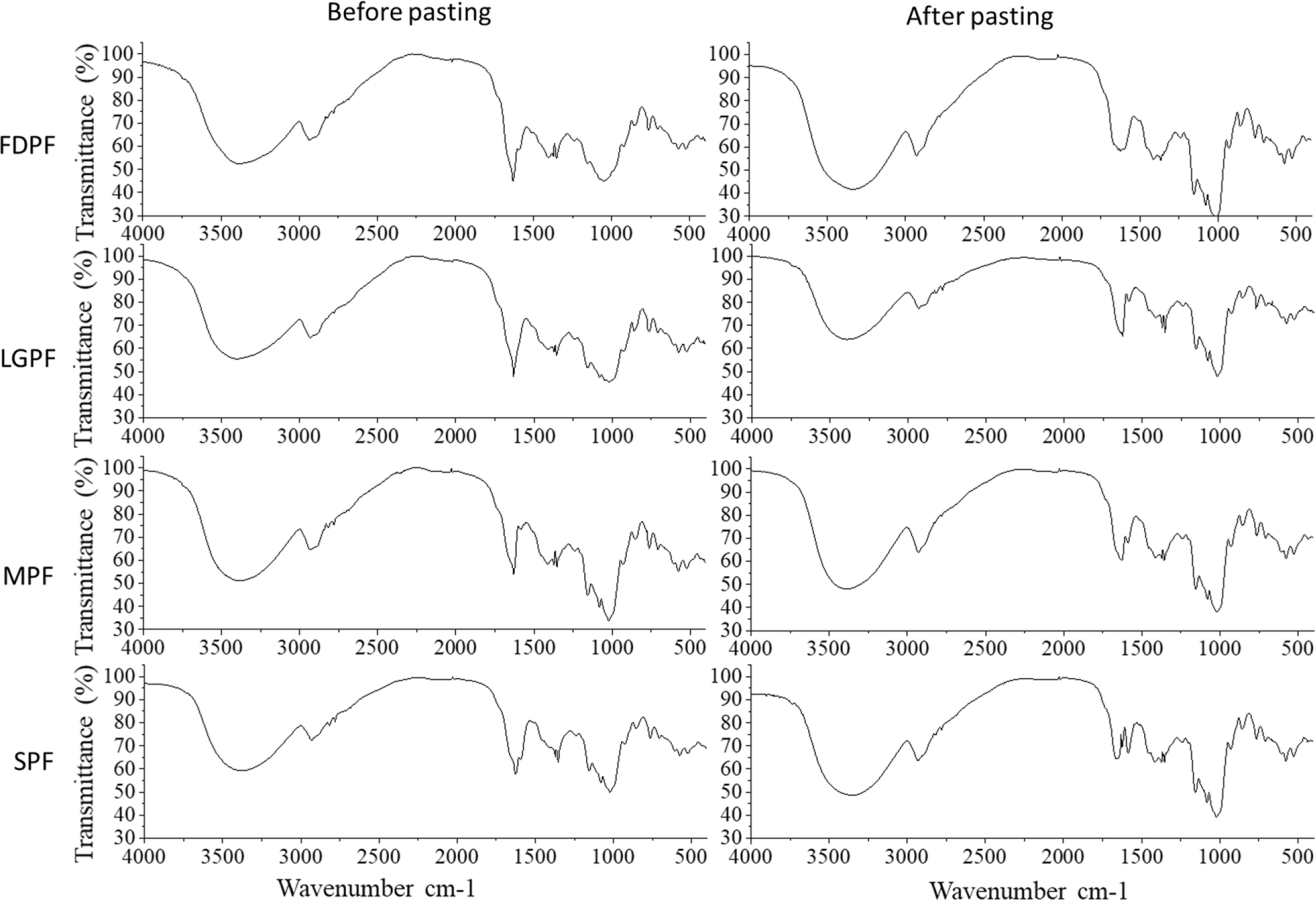
Vibrational spectra of potato flour and their paste

FTIR spectra for the potato flour and their paste were all similar in shape but not intensity (Fig. 4), indicating that heating and shearing had effect on long-range ordering and crystallinity. The range at 3800–3000 cm^−1^ of the IR spectra represent ν-stretching of O–H [15]. For FDPF and SPF, the combination of temperature and shear stress increased the IR intensity of the peaks in the range at 3800–3000 cm^−1^. However, this was opposite for LGPF. ν(O–H) of SPF was the weakest than other potato flour. The IR intensity of the peaks centered at ~ 1655 cm^−1^ represent δ-deformation of O–H [15]. For FDPF, LGPF and SPF, the combination of temperature and shear stress had negative effect on δ-deformation of O–H. The IR intensity of the peaks centered at ~ 1082 cm^−1^ represent the bending of C–H [15]. For FDPF and SPF, the combination of temperature and shear stress increased the IR intensity of the peaks centered at ~ 1082 cm^−1^. There was no significant difference in IR spectra between MPF and its paste.

## Conclusion

The effect of temperature and shear on the viscoelastic properties and molecular structure of potato flour prepared by different methods was investigated. The effect of high temperature shearing on the binding of covalent bonds in potato flour was different. Results of FTIR showed that the combination of temperature and shear stress had positive effect on ν-stretching of O–H in FDPF and SPF. However, the combination play negative role on ν-stretching of O–H in LGPF. ν(O–H) of SPF was the weakest than other potato flour. For FDPF, LGPF and SPF, high temperature shear had negative effect on δ-deformation of O–H. For FDPF and SPF, high temperature shear play positive role on bending of C–H. Results of XRD showed that the crystalline properties of different potato flour after shearing at high temperature were the same. In addition, low gelatinization potato flour presented a crystalline structure or strong internal order. Texture results showed that FDPF and LGPF had good gel stability, while MPF and SPF gel had poor stability. LGPF had good retrogradation after hot shearing, which was more conducive to the formation of hot-processed products.

## Materials and methods

### Materials

Potato tubers were purchased from Taste From The Field (Shouguang, Shandong province, China). Citric acid (analytical reagents) was purchased from Beijing Chemical Works (Beijing, China).

### Preparation of potato flour

Some fresh potatoes were cleaned and steamed for 40 min, peeled artificially, and then dryed by hot air (65 °C) in a Natural convection oven (DHG-9055A, Bluepard, China) for 12 h. In addition, some tubers were peeled using a fruit parer. Immediately, the peeled potatoes were kept in water to prevent browning. Following, the tubers were cut into slices (2.5 mm thick) using a fruit slicer. Finally, some potato slices were freeze-dried for 48 h, the others were soaked in a compounded anti-browning agents containing 0.4% citric acid for 30 min to avoid enzymatic browning and dried by hot air in Natural convection oven (DHG-9055A, Bluepard, China) for 12 h, or heated for 4 min with a microwave (P70D20AP-TE(W0), 700W, Galanz, China) and dried by hot air in natural convection oven (DHG-9055A, Bluepard, China) for 12 h. After microwave for 2 min, potato slices were turned over before microwave for the remaining 2 min.

The dried potato mass or slices were smashed by a Mill (600A, RONGHAO, China) for 15 s and sieved with 60 mesh. According to Zhang et al.’s [16] method, potato flour was placed in a constant temperature and humidity sealed container to regulate humidity, and the final moisture content was about 14%.

### Degree of gelatinization

The DG was determined according to the Chemical Industry Standards of the People’s Republic of China (HG/T 3932-2007). Firstly, two laboratory samples (1 g each) were weighted and placed in 150 mL conical bottles and labeled A and B, respectively. An empty 150 mL triangular bottle was regarded as control and marked C. 40 mL phosphoric buffer (pH = 6.8) was added to A, B and C. A was heat in boiling water bath for 30 min and then cooled down to below 60 °C. 5  mL β-amylase solution (6 g β-amylase was dissolved in 100 mL phosphoric buffer and its enzymatic activity was greater than 80,000 units) was added to these three samples, respectively. Secondly, A, B and C were put in a water bath (40 °C) for 1 h and they were shook once every 15 min. 2 mL sulfuric acid solution (10%, V/V) and 2 mL sodium tungstate solution (120 g/L) were added to A, B and C, and then shake well and diluted to 100 mL with distilled water. Solutions in 100 mL volumetric bottles were filtered with neutral filter paper. Thirdly, 5 ml filtrate was added to a 150 mL conical bottle, then 15 mL 0.1 mol/L alkaline potassium ferricyanide solution was added and boiled in water bath for 20 min. After cooling to room temperature, the samples were added 25 mL acetate solution (70 g potassium chloride and 40 g zinc sulfate are dissolved in distilled water, heated to complete dissolution, cooled to room temperature, mixed with 200 mL glacial acetic acid and diluted to 1000 mL) and 5 mL 100 g/L potassium iodide solution in turn. Immediately, the solution was titrated to light yellow with 0.1 sodium thiosulfate solution and added three drops of 10 g/L starch indicator solution, then titrated until the blue disappeared. The DG of the sample was calculated according to the following formula:

1 $${\text{DG}} = \left( {\left( {{\text{P}} - {\text{m}}} \right)*100} \right)/\left( {{\text{P}} - {\text{n}}} \right)$$

where DG, degree of starch gelatinization (%); P, volume consumption of sodium thiosulfate in blank experiment (mL); m, volume consumption of sodium thiosulfate in complete gelatinization experiment (mL); n, volume consumption of sodium thiosulfate in sample experiment (mL). Each value was mean of three replicates.

### Amylose content, swelling power and light transmittance

Amylose contents were determined by iodine colorimeter at 620 nm using a potato starch standard mixture [17].

The swelling properties were measured following the procedure by Zhang and Ma et al. with some modification [16, 18]. 0.25 g samples and 7 mL distilled water were added to the 15 mL centrifuge tube, which would successively experience vortex oscillation, water bath oscillating at 70 °C for 10 min, vortex oscillation, boiling water bath for 10 min, ice-bath for 5 min, centrifugation (4000 r/min, 10 min). The procedure was repeated two times. The data of swelling properties was calculated according to the following formula.

$${\text{Swelling}}\;{\text{power}} = \frac{{{\text{Weight }}\;{\text{of}}\;{\text{the}}\;{\text{wet}}\;{\text{precipitate}} \;({\text{g}})}}{{{\text{Weight}}\;{\text{of}}\;{\text{the}}\;{\text{dried}}\;{\text{sample}}\; ({\text{g}})}}.$$

The light transmittance was determined according to the method of Heo [19]. 1% (g/mL) sample suspension was placed in a boiling water bath for 30 min, with vortexing every 5 min. After cooling to room temperature, the light transmittance was read at 620 nm against a distilled water blank.

### Differential scanning calorimetry

The gelatinization of potato flour was analyzed by differential scanning calorimetry (DSC-60, SHIMADZU, Japan) under ultra-high purity nitrogen atmosphere according to a conventional procedure stated by Cui’s team [20]. 3 mg sample (dry base) and 6 μL deionized water were placed in aluminum crucible. An empty crucible was used for the control. Sealed container balanced at room temperature for 12 h to hydrate prior to testing. The specimens were heated at a constant rate of 10 °C min^−1^ from 30 to 110 °C to perform the gelatinization process. Gelatinization enthalpy change (ΔH_G_) was determined according to areas below the curves and was shown in the form of mJ.

### Particle size and distribution

Particle size distribution of potato flour was analyzed using a Laser particle size analyzer (LS230, Beckman, USA). Each value was mean of three replicates.

### Structural characterization

According to a conventional procedure stated previously [21], the sample was mounted on an aluminum stub using double-sided stick tape, and then examined at a JCM-6000 scanning electron microscope (JEOL Ltd, Japan) at an acceleration voltage of 10 kV. The morphology and dispersion of particles in suspensions were determined by optical microscopy according to the method described by Zhao et al. [22]. The glass substrates that prepared with the 5% (w/v) suspension was observed under an optical microscope (10× objective).

### Pasting properties

The pasting properties of potato flour were measured in accordance with the method as defined by the published literature with rapid viscosity analyzer (RVA, Newport Scientific Inc., Australian) [23]. 25 mL distilled water and 3 g sample were added to RVA test aluminum barrel. The speed of 160 r/min, the designed temperature program (50 °C–95 °C–50 °C), and the determination time of 13 min were applied to the operation of the equipment. The obtained potato flour pastes were stored at 4 °C for 24 h to enable gel structure to form. Additionally, Fourier-transform infrared spectroscopy (FTIR), X-ray diffraction (XRD) and texture measurements of the potato flour paste obtained immediately after gelatinization were performed.

### Textural properties

The textural properties were measured following the procedure by Li et al. [24] with some modification, and the procedure was repeated five times. Adjusting 1 mm/s of test speed and 30% of compression for a test.

### X-ray diffraction

According to the procedure stated previously [25], XRD patterns of potato flour and their paste were prepared using an X-ray diffractometer (SmartLab, Rigaku, Japan) equipped with Cu radiation at a wavelength of 1.5406 Å. Measurements were obtained at room temperature with a scanning rate of 0.02°/s and a diffraction angle range of 5 to 80° (2-Theta range), where theta is the angle of incidence of the X-ray beam on the sample. The diffraction patterns were analysed using Origin 9.

### FTIR

FTIR spectra were collected for potato flour and their paste using a FTIR spectrometer (SPECTRUM 100, PerkinElmer, American). 1–2 mg samples were blended with 200 mg KBr and pressed into tablets before measurement. Spectra were collected at a resolution of 4 cm^−1^ and at an average of 4 scans per sample.

### Statistical analysis

All experiments were replicated at least thrice. All test data were statistically analyzed by one-way ANOVA using SPSS 19.0, and expressed as mean values ± standard deviations. Independent T-test and Duncan’s multiple range test was used to determine significant differences. Differences were regarded as significant at 95% (p < 0.05).

## Data Availability

The dataset supporting the conclusion of this article is included within the article.

## Abbreviations

DSC: Differential scanning calorimetry
XRD: X-ray diffraction
FTIR: Fourier-transform infrared spectroscopy
DG: Degree of starch gelatinization
FDPF: Freeze-dried potato flour
LGPF: Low gelatinization potato flour
MPF: Microwave potato flour
SPF: Steamed potato flour
